# Pseudohypoparathyroidism type 1a with hypomethylation at the responsible differentially methylated region for Beckwith-Wiedemann syndrome

**DOI:** 10.1186/1687-9856-2015-S1-O51

**Published:** 2015-04-28

**Authors:** Yoshiaki Ohtsu, Kanako Kurata, Mai Takahashi, Takanori Kowase, Hirokazu Arakawa, Shinichiro Sano, Masayo Kagami

**Affiliations:** 1 Gunma University Graduate School of Medicine, Department of Pediatrics, Maebashi, Japan; 2 National Research Institute for Child Health and Development, Department of Molecular Endocrinology, Tokyo, Japan

## Aim

It was previously reported that several patients with pseudohypoparathyroidism type 1b (PHP-1b) have a more generalised imprinting defect. However there was no report that a patient of PHP-1a has any generalised imprinting defects. Here we aim to report a case of PHP-1a with hypomethylation at the Kv-differentially methylated region (DMR) (11p15.5), responsible for Beckwith-Wiedemann syndrome.

## Method

A 6-year-old girl admitted to our hospital because of tetany due to hypocalcemia.

## Result

She was a full-term infant and was delivered after an uncomplicated pregnancy. She did not show macrosomia, omphalocele or macroglossia. The neonatal period was uncomplicated. Her TSH level was in the normal range on neonatal screening. Her parents and younger sister did not show any visible sign of Albright’s hereditary ostedystrophy. At 5 months old, she was severely obese and clinical follow-up was started. At 2 years old, her serum TSH level was 11.8 IU/l and free T4 was 0.4 ng/dl. Her serum calcium level and phosphate level were within normal ranges. She was diagnosed as having hypothyroidism and levothyroxine replacement therapy was initiated. Her body weight then gradually decreased. She did not suffer from mental retardation.

At 6 years old, she was admitted to our hospital because of tetany when she had an upper urinary tract infection. On admission to our hospital, she presented with a round chubby face, short neck and obesity. Radiography indicated short metacarpals, which mainly affected the fourth digit. Computed tomography indicated ectopic ossifications at putamen. Biochemistry revealed hyperphosphatemia and increased serum concentrations of parathyroid hormone. Urinary excretions of cAMP and phosphate did not increase after intravenous infusion of PTH, suggesting PTH resistance in the kidney. Molecular analysis revealed a maternally inherited 2-Mb deletion of 20q13.3- 20q13.32 including the GNAS locus and the adjacent STX16 locus. Furthermore, a loss of maternal methylation on the Kv-DMR region was detected. A specific diagnosis was made of PHP-1a in the presence of Beckwith-Wiedemann syndrome. She was treated with 1,25-dihydroxyvitamin D3 [1,25(OH)_2_D3] (0.02 µg/kg/day) and calcium lactate (0.1 g/kg/day) was initiated. By this treatment, hypocalcemia was improved. Serum intact-PTH level has gradually decreased to 100-200 pg/ml. Her urinary calcium excretion has remained below 0.2 (calcium (mg/dl)/creatinine (mg/dl).

## Conclusions

This is the first case report to describe a combination of PHP1a with a generalised imprinting defect, and their co-existence should be considered and further investigated.

Written informed consent was obtained from the patient for publication of this abstract and any accompanying images. A copy of the written consent is available for review by the Editor of this journal.

